# Protein, iron, and meat consumption and risk for rheumatoid arthritis: a prospective cohort study

**DOI:** 10.1186/ar2123

**Published:** 2007-02-08

**Authors:** Elizabeth Benito-Garcia, Diane Feskanich, Frank B Hu, Lisa A Mandl, Elizabeth W Karlson

**Affiliations:** 1 Section of Clinical Sciences, Division of Rheumatology, Immunology and Allergy, Department of Medicine, Brigham & Women's Hospital, Francis Street, Boston, Massachusetts 02115, USA; 2 BioEPI Clinical and Translational Research Center, Taguspark, Núcleo Central,232 2740-122 Oeiras, Portugal; 3 Channing Laboratory, Department of Medicine, Brigham and Women's Hospital and Harvard Medical School, Longwood Avenue, Boston, Massachusetts 02115, USA; 4 Department of Nutrition, Harvard School of Public Health, Huntington Avenue, Boston, Massachusetts 02215, USA; 5 Rheumatology Clinical Research Center, Hospital for Special Surgery, East 70th Street, New York, New York 10021, USA

## Abstract

A recent prospective study showed that higher consumption of red meat and total protein was associated with increased risk for inflammatory polyarthritis. We therefore prospectively examined the relationship between diet (in particular, protein, iron, and corresponding food sources) and incident rheumatoid arthritis (RA) among 82,063 women in the Nurses' Health Study. From 1980 to 2002, 546 incident cases of RA were confirmed by a connective tissue disease screening questionnaire and medical record review for American College of Rheumatology criteria for RA. Diet was assessed at baseline in 1980 and five additional times during follow up. We conducted Cox proportional hazards analyses to calculate the rate ratio of RA associated with intakes of protein (total, animal, and vegetable) and iron (total, dietary, from supplements, and heme iron) and their primary food sources, adjusting for age, smoking, body mass index, and reproductive factors. The multivariate models revealed no association between RA and any measure of protein or iron intake. In comparisons of highest with lowest quintiles of intake, the rate ratio for total protein was 1.17 (95% confidence interval 0.89–1.54; *P *for trend = 0.11) and for total iron it was 1.04 (95% confidence interval 0.77–1.41; *P *for trend = 0.82). Red meat, poultry, and fish were also not associated with RA risk. We were unable to confirm that there is an association between protein or meat and risk for RA in this large female cohort. Iron was also not associated with RA in this cohort.

## Introduction

Rheumatoid arthritis (RA) is associated with both genetic and environmental factors [1-7], but studies of dietary risk factors have been inconclusive [8]. Studies of diet and risk for RA offer the potential to identify modifiable factors and so prevent RA in high-risk patients; they may also provide insights into disease pathogenesis.

Buchanan and Laurent [9] implicated diets high in protein in the etiology of RA. Furthermore, low-protein diets may improve RA symptoms [10-13]. In ecologic studies, the prevalence of RA is higher in countries with greater consumption of red meat [14]. More recently, Pattison and colleagues [15] reported the first prospective investigation of red meat and risk for inflammatory polyarthritis (IP) and concluded that higher intakes of both red meat and protein increased the risk for IP, whereas iron – another nutrient component of meat – exhibited no association. The authors acknowledged that it remained unclear whether the observed associations were causative or whether meat consumption was a marker for other lifestyle factors.

To examine this issue further, we prospectively assessed risk for RA in relation to intakes of protein, iron, and meat in women in the Nurses' Health Study (NHS). We examined these intakes with further classifications into animal and vegetable protein; dietary, supplemental, and heme iron; and red meat, poultry, and fish.

## Materials and methods

The NHS was established in 1976 when 121,700 female registered nurses (98% white), aged 30–55 years and residing in one of 11 US states, completed and returned the initial NHS mailed questionnaire on their medical history and lifestyle. Every 2 years, follow-up questionnaires have been sent to obtain up-to-date information on risk factors and to identify newly diagnosed diseases. Deaths are reported by family members or by the postal service in response to the follow-up questionnaires. In addition, we use the National Death Index to search for nonrespondents who might have died in the preceding interval. By comparing deaths ascertained from independent sources, we estimate that we have identified at least 98.2% of deaths occurring in the cohort [16].

The Partners HealthCare Institutional Review Board approved all aspects of this study, and all participants gave informed consent before they were entered into the study.

### Ascertainment of rheumatoid arthritis cases

As previously described [17], self-reports of RA were confirmed using the Connective Tissue Disease Screening Questionnaire [18] and by medical record review for American College of Rheumatology (ACR) criteria for RA [19], conducted by two rheumatologists. We confirmed 807 cases of incident RA from 1976 to 2002.

### Study population

For all analyses, we excluded the following: prevalent RA cases that were diagnosed before June 1980; RA cases with missing date of diagnosis; women who reported RA or connective tissue disease but in whom the diagnosis of RA was not confirmed by medical record review; nonresponders to the semiquantitative Food Frequency Questionnaire (FFQ) in 1980 (the baseline for this analysis); and participants with an unacceptable FFQ (<500 kcal/day or >3,500 kcal/day, accounting for approximately 4% of returned dietary questionnaires). Women were also censored during follow up when they failed to respond to any subsequent biennial questionnaire, because incident RA could not be identified in these cases. Thus, the final group studied included 82,063 women who were followed from 1980 until 2002 and 546 cases of incident RA who met the inclusion criteria, with a total of 1,668,894 person-years of follow up.

### Assessment of dietary intake

Dietary intake was assessed in 1980, 1984, 1986, 1990, 1994, and 1998 using a semi-quantitative FFQ. In 1980, a total of 98,462 (81%) of the participants completed the FFQ and the completion rate has remained at about 80% during follow up. The initial FFQ contained 61 food items, but it has been expanded over the years such that 147 foods appeared on the 1998 questionnaire, including nine items for red meat (beef, pork, and lamb), four items for poultry (chicken and turkey), and four items for fish. For each food, participants reported their frequency of consumption of a specified serving size using nine frequency categories, ranging from never to six or more per day.

The validity and reproducibility of the FFQ for nutrients [20] and foods [21] have been documented elsewhere. Intakes calculated from the 1980 FFQ were found to be reasonably correlated with those from four 1-week diet records collected over 1 year among 173 NHS participants [20,22]. The Pearson coefficients were 0.47 for total protein, 0.55 for total iron [20], and 0.38 for meat [21].

In this analysis, we examined associations between risk for RA and intakes of the following individual nutrients and components: total protein, animal protein, vegetable protein, total iron, dietary iron (from food sources), supplemental iron (from multivitamins and supplements), and heme iron (the iron with the highest bioavailability). We also examined meat, poultry, and fish (the primary food sources of protein and iron). At the 1998 dietary assessment in this cohort, 19% of protein came from red meat, 14% came from poultry, and 7% from fish. Heme iron also came primarily from the consumption of red meat (28%), poultry (24%), and fish (15%). Supplements contributed 25% of the total iron intake in this cohort.

### Assessment of nondietary factors

Age, body mass index (weight [in kilograms] divided by height [in meters]^2^), and smoking status were updated every 2 years with information from the biennial questionnaires. Other factors were reported once: age at menarche in 1976, total months of breastfeeding for all children in 1986, and regularity of menses from age 20 to 35 years (very regular, usually regular, usually irregular, and very irregular) in 1982.

### Statistical analyses

The number of person-years of follow up was ascertained based on the interval between the date of return of the 1980 questionnaire and the date of diagnosis of RA (as defined in the medical record), death, the end of the study period (1 June 2002), or loss to follow up (defined as no further return of questionnaires) for each participant.

Nutrient and food intakes were categorized into quintiles, and incidence rates for RA were calculated by dividing the number of incident cases by the number of person-years in each quintile of dietary exposure. Rate ratio (RRs) were calculated by dividing the incidence rates in the higher quintiles by the corresponding rate in the reference (lowest) quintile. Age-adjusted and multivariate RRs were estimated using Cox proportional hazards models adjusting for age (continuous variable) and other potential counfounders. We controlled for the following variables because they have either been shown to be associated with RA or were found in this study to be potential confounders: body mass index (categorized as <22, 22 to 24.9, 25 to 29.9, 30 to 34.9, and ≥35 kg/m^2^), smoking status (never, past, or present), and total lifetime breastfeeding history (nulliparous, parous, and breastfeeding for 0, 1 to 11, ≥12 total months). In addition, we controlled for total energy to reduce measurement error due to general over-reporting or under-reporting of food items [23]. Age at menarche and regularity of menses were not retained as covariates. For all RRs, we calculated the 95% confidence interval (CI). All *P *values were two-tailed, and *P *< 0.05 was considered to be statistically significant. Tests for trend were conducted by assigning the median value for each quintile of nutrient and food intake, modeling this variable as a continuous variable.

Nutrient intakes were energy-adjusted using the multivariate residual method [20]. In order to represent the long-term dietary patterns of individual women, our primary analysis used cumulative average food and nutrient intakes from all available dietary questionnaires up to the start of each 2-year interval [24]. For example, the 1980 diet was related to RA incidence during the period from 1980 to 1984; the average of the 1980 and 1984 diets was related to RA incidence during the period from 1984 to 1986; the average of the 1980, 1984, and 1986 diets was related to the RA incidence during the period between 1986 and 1990, and so on, through to 2002.

## Results

Age standardized characteristics of the study population in 1990 according to intakes of total protein and heme iron are shown in Table 1. The 1990 time point was chosen because it represents the approximate mid-point of follow up. Body mass index was higher among women in the highest consumption categories of total protein and heme iron. Women with the lowest protein and highest heme iron consumptions were more likely to smoke and, if parous, they were less likely to have breastfed for a total of 12 months or more. Higher total protein intakes were associated with higher heme iron intakes.

In the age-adjusted model, higher total protein intake was associated with greater risk for RA (quintile 5 [89.0 g/day] versus quintile 1 [60.8 g/day]: RR 1.23, 95% CI 0.94–1.61; *P *for trend = 0.04), but this association was attenuated and the test for trend was no longer significant in the multivariate model (RR 1.17, 95% CI 0.89–1.55; *P *for trend = 0.12; Table 2). Neither the animal nor vegetable component of protein exhibited any relation to risk for RA. We also did not observe any association with total iron intake (RR 1.00, 95% CI 0.74–1.36 for the highest versus lowest quintile) or with its components of dietary iron, supplemental iron, and heme iron.

No significant associations were observed between the incidence of RA and consumption of total meat, red meat, poultry, or fish (Table 3). For total meat, which included red meat and poultry, the multivariate RR was 0.91 (95% CI 0.67–1.23) in the highest (2.54 servings/day) versus lowest (0.82 servings/day) quintiles of intake. More detailed analyses of individual foods that contribute to each of these major food groups also exhibited no association with RA.

To avoid confounding by indication (for example, dietary changes occurring after RA symptom onset), we also performed analyses in which dietary variables were updated only until the date of first symptom of RA, rather than until the date of RA diagnosis. We also performed lagged analyses such that the dietary intakes associated with RA cases were assessed at least 4 years before the date of diagnosis. In order to account for possible influence of recent dietary intake, we also examined our exposures based on the most recent dietary measures, rather than using long-term average intakes. The results revealed no associations with the nutrient or food exposures.

## Discussion

In this large prospective cohort study involving women, we observed no significant association between protein or iron intakes and risk for RA, including specific analyses of animal and vegetable protein, heme iron, and iron from foods and from supplements. Furthermore, no associations were observed between the primary food sources of these nutrients, namely red meat, poultry, and fish.

Our results differ from those of a nested case-control study [15] that reported increased risk of IP with greater consumption of protein and red meat. Pattison and coworkers [15] studied dietary intake and risk for IP between 1993 and 2002, within a prospective population-based study of cancer incidence in Norfolk, England (European Prospective Investigation of Cancer Incidence [EPIC]). In their study they compared 88 patients with IP, identified by linkage with the Norfolk Arthritis Register (a primary care-based inception study of IP), with 167 age-matched and sex-matched control individuals from EPIC who had remained free from IP during the follow-up period. Although the study did not analyze subtypes of protein, animal and vegetable protein, it did analyze the food sources that contribute to each of these categories. The investigators reported an increased risk for IP with greater protein comsumption (>75.3 g/day versus <62.4 g/day: adjusted odds ratio [OR] 2.9, 95% CI 1.1–7.5) and no association with iron. In contrast to our findings, the study by Pattison and coworkers indicated that individuals with the highest level of consumption of red meat (>58.0 g/day versus <25.5 g/day: adjusted OR 1.9, 95% CI 0.9–4.0) and red meat combined with meat products (for instance, sausage and ham; >87.8 g/day versus <49.0 g/day: adjusted OR 2.3, 95% CI 1.1–4.9) were at increased risk for IP.

The discrepancy between the findings of that study and ours could be attributed to methodologic differences. First, the EPIC study assessed dietary intake once, using a 7-day food diary, whereas we used semiquantitative FFQ assessed repeatedly. The FFQ consists of two components [25]: a food list and a frequency response section for individuals to report how often each food was eaten over the previous year. The 7-day food diary consists of a detailed listing of all foods consumed by an individual on 1 day or more [26]. Food intake is recorded by the individual at the time when the foods are eaten, which has the advantages of relying less on memory and permitting direct assessment of portion sizes. In comparison, the FFQ suffers the disadvantages of restrictions imposed by a fixed list of foods, memory, perception of portion sizes, and interpretation of questions. Dietary records provide more precise quantification of foods consumed, but they only reflect short-term diet, because only a limited number of days of diet records are used. Results of validation studies demonstrate greater correlation of blood levels of certain nutrients with 7-day diet diaries than with FFQ findings [27].

However, our objective was to assess long-term dietary exposures. Therefore, we cumulatively averaged and updated dietary intake assessed six different times over the 22-year period of follow up, which is known to reduce random error in long-term dietary measurement, rather than relying upon one assessment at baseline. Furthermore, results of analyses of more recent diet were consistent with analyses of cumulative diet. Even if absolute measures are not precise, the FFQ is able to rank respondents into higher and lower categories of intake. We energy-adjusted nutrient intakes in order to account for differences due to under-reporting or over-reporting on the FFQ.

Bingham and coworkers [28] demonstrated a strong association between diet and cancer using 7-day diaries but a modest relationship when the FFQ was used, and they suggested that this pattern might also be seen in other studies analyzing the association of diet and chronic diseases. However, previous studies undertaken in the Nurses' Health Study cohort and others that used the FFQ demonstrated associations between meat and protein and breast cancer, colorectal cancer, lymphoma, coronary heart disease, diabetes, and gout [29-35].

Finally, it is possible that dietary protein intake differs between the USA and the UK. However, comparisons of the median intake of total protein and total iron in the quintiles used in the present study (Table 2) with the tertiles of intake in the EPIC study [15] demonstrate that the range and categories of intake in the two studies were similar.

A second difference between our study and the EPIC study was that we identified individuals with RA rigorously using the ACR criteria, in which at least four out of seven criteria had to be satisfied in order for a participant to be considered a case. In contrast, the outcome considered by Pattison and colleagues [15] was the presence of IP, which is defined as inflammation affecting two or more peripheral joints and persisting for 4 weeks or longer. Within 5 years, 60% of IP patients satisfy ACR criteria for RA [36].

Third, discrepancies between our study and the EPIC study might be related to differences in sex, because our study included women only whereas the EPIC study [15] included men and women. It is also possible that the discrepant findings resulted from socioeconomic differences; well educated nurses were enrolled in our study, whereas the EPIC cohort included diverse population-based cases and controls.

Strengths of our study include the large number of incident cases of RA, the repeated prospective assessment of exposures, and the lengthy follow-up period. The validation of self-reported RA through medical record review rather than by physical examination is a potential weakness of the study. However, 82% of the RA cases were diagnosed by ACR members, which adds support to the validity of the diagnoses. There is potential for misclassification of RA cases as noncases when diagnosis relies solely upon medical record documentation. Therefore, those women who self-reported RA or other connective tissue diseases in whom the diagnosis of RA was not confirmed by medical record review were excluded from the analyses. It is possible that the null results from this study are due to unmeasured confounding (for example, socioeconomic status), although there are no strong risk factors for RA that could account for attenuation of a true association. Finally, although the participants in the present do not represent a random sample of women living in the USA, it is unlikely that the biologic relationships among these women will differ from those among women in general.

## Conclusion

No clear associations were observed between dietary protein, iron, or meat, including red meat, and risk for RA in this large prospective cohort of women.

## Abbreviations

ACR = American College of Rheumatology; CI = confidence interval; FFQ = Food Frequency Questionnaire; IP = inflammatory polyarthritis; OR = odds ratio; RA = rheumatoid arthritis; RR = rate ratio.

## Competing interests

The authors declare that they have no competing interests.

## Authors' contributions

EB-G contributed to the study concept and design, data collection and analyses, and manuscript writing and editing. DF contributed to the data analyses and statistical support, as well as to the manuscript writing and editing. FB contributed to the concept and design, and to the manuscript writing and editing. LAM contributed to data collection and manuscript writing and editing. EWK obtained the funding and contributed to the concept and design, data collection and analyses, and manuscript writing and editing.

## Figures and Tables

**Table 1:** Age-standardized characteristics of the subjects in the Nurses' Health Study in 1990

	Total protein (g/day)			Heme iron (g/day)		
Quintile	1	3	5	1	3	5

Intake (mean)	60.1	74.1	90.7	0.77	1.20	1.81
Number of women	15,836	15,822	15,824	15,843	15,996	15,649
Age (mean; years))	56.4	56.3	57.1	57.3	56.4	56.3
Body mass index (mean; kg/m^2^)	24.7	25.5	27.1	24.6	25.8	26.6
Current smoker (%)	23	16	14	14	17	21
Age at menarche (mean; years)	12.6	12.5	12.3	12.5	12.5	12.4
Breastfeeding ≥12 months among parous women (%)	14	17	17	18	17	13
Very regular menses (%)^a^	53	56	54	56	57	50
Mean nutrient intakes						
Protein (g/day)	-	-	-	67.1	74.6	82.5
Animal protein (g/day)	42.4	55.8	73.0	47.3	56.5	66.7
Vegetable protein (g/day)	17.7	18.2	17.7	19.8	18.1	15.7
Heme iron (g/day)	0.98	1.25	1.50	-	-	-
Total iron (g/day)	14.0	15.5	16.5	15.8	15.6	14.8

A total of 79,173 are included, and findings are presented according to lowest, middle, and highest consumption categories of total protein and total heme iron. Values are presented as mean or percentage of the population within each category, and are standardized to the age distribution of the study population over follow up from 1980 to 1990. The lowest, middle, and highest categories are three of the five consumption categories used in Tables 2 to 4. ^a^At age 20 to 35 years.

**Table 2:** Rate ratios of rheumatoid arthritis by protein and iron intakes among women in the Nurses' Health Study, 1980 to 2002

Nutrients	Quintile	Median intake	Number of cases	Age-adjusted^a ^rate ratio (95% CI)	Multivariate^b ^rate ratio (95% CI)
Total protein^c^	1	60.8 g/day	95	1.00	1.00
	2	68.4 g/day	109	1.12 (0.86–1.48)	1.11 (0.84, 1.47)
	3	73.9 g/day	89	0.90 (0.67–1.20)	0.90 (0.67, 1.21)
	4	79.5 g/day	133	1.35 (1.04–1.76)	1.34 (1.03, 1.76)
	5	89.0 g/day	120	1.23 (0.94–1,61)	1.17 (0.88, 1.55)
				*P *for trend = 0.04^d^	*P *for trend = 0.12^d^
Animal protein^e^	1	42.5 g/day	101	1.00	1.00
	2	50.4 g/day	99	0.97 (0.74–1.28)	0.93 (0.71–1.24)
	3	56.1 g/day	104	1.02 (0.78–1.34)	1.03 (0.78–1.36)
	4	62.3 g/day	125	1.23 (0.95–1.60)	1.21 (0.92–1.59)
	5	72.9 g/day	117	1.13 (0.87–1.49)	1.13 (0.84–1.50)
				*P *for trend = 0.08^d^	*P *for trend = 0.14^d^
Vegetable protein^e^	1	12.2 g/day	111	1.00	1.00
	2	15.5 g/day	95	0.81 (0.61–1.06)	0.83 (0.62–1.11)
	3	17.5 g/day	101	0.82 (0.62–1.08)	0.89 (0.66–1.21)
	4	19.3 g/day	127	1.04 (0.79–1.36)	1.14 (0.84–1.55)
	5	22.2 g/day	112	0.90 (0.67–1.20)	1.02 (0.74–1.41)
				*P *for trend = 0.89^d^	*P *for trend = 0.36^d^
Total iron intake (diet and supplements)^f^	1	8.00 mg/day	101	1.00	1.00
	2	10.0 mg/day	110	1.01 (0.76–1.34)	1.01 (0.76–1.35)
	3	11.8 mg/day	111	1.00 (0.75–1.33)	0.99 (0.74–1.33)
	4	15.2 mg/day	117	1.12 (0.84–1.49)	1.10 (0.81–1.48)
	5	24.4 mg/day	107	1.00 (0.75–1.34)	1.00 (0.74–1.36)
				*P *for trend = 0.81^d^	*P *for trend = 0.98^d^
Dietary iron^g^	1	8.10 mg/day	117	1.00	1.00
	2	9.78 mg/day	80	0.90 (0.67–1.20)	1.01 (0.76–1.35)
	3	10.6 mg/day	113	1.01 (0.77–1.34)	0.99 (0.74–1.33)
	4	11.9 mg/day	114	1.10 (0.83–1.44)	1.10 (0.81–1.48)
	5	14.4 mg/day	122	1.20 (0.92–1.56)	1.00 (0.74–1.36)
				*P *for trend = 0.21^d^	*P *for trend = 0.92^d^
Iron from supplements^g^	1	0.00 mg/day	371	1.00	1.00
	2	2.08 mg/day	41	0.73 (0.52–1.03)	0.79 (0.56–1.11)
	3	10.0 mg/day	134	0.92 (0.75–1.12)	0.94 (0.77–1.16)
				*P *for trend = 0.27^d^	*P *for trend = 0.68^d^
	1	0.80 mg/day	99	1.00	1.00
Heme iron intake^h^	2	1.04 mg/day	121	1.21 (0.92–1.57)	1.16 (0.88–1.52)
	3	1.23 mg/day	106	1.08 (0.82–1.42)	0.99 (0.74–1.31)
	4	1.46 mg/day	113	1.08 (0.82–1.43)	0.99 (0.74–1.32)
	5	1.90 mg/day	107	1.18 (0.89–1.57)	0.96 (0.70–1.31)
				*P *for trend = 0.49^d^	*P *for trend = 0.51^d^

In all, 82,063 women were included and there were 546 incident cases of rheumatoid arthritis (RA). Diet was assessed in 1980, 1984, 1986, 1990, 1994, and 1998. Nutrient intakes were cumulatively updated over follow up. Total intakes include multivitamins and supplements. Dietary intakes are from food sources only. ^a^The age-adjusted models were adjusted for the total energy intake as well as age. ^b^The multivariate models were adjusted for age, body mass index, smoking, and total lifetime breastfeeding. ^c^Protein was adjusted for total iron as well as for the same variables adjusted for in all the multivariate models. ^d^All *P *values for trend were calculated with median intake of each nutrient in each quintile as a continuous variable. ^e^Animal and vegetable protein were adjusted for each other, total iron, and for the same variables adjusted for in all the multivariate models. ^f^Total iron was adjusted for total protein as well as for the same variables adjusted for in all the multivariate models. ^g^Dietary and supplemental iron were adjusted for each other, total protein, and the same variables adjusted for in all of the multivariate models; supplemental iron is given in tertiles. ^h^Heme iron models are additionally adjusted for total protein. CI, confidence interval.

**Table 3:** Rate ratios for rheumatoid arthritis across quintiles of meat, poultry, and fish consumption among women in the Nurses' Health Study, 1980 to 2002

	Quintile	Median intake (servings/day)	Number of cases	Age-adjusted rate ratios (95% CI)	Multivariate^a ^rate ratios (95% CI)
Total meat (red meat and poultry)	1	0.82	118	1.00	1.00
	2	1.20	107	0.91 (0.70–1.18)	0.89 (0.68–1.17)
	3	1.52	127	1.08 (0.84–1.39)	1.09 (0.84–1.42)
	4	1.89	94	0.79 (0.60–1.04)	0.81 (0.61–1.08)
	5	2.54	100	0.86 (0.65–1.12)	0.91 (0.67–1.23)
				*P *for trend = 0.18^b^	*P *for trend = 0.55^b^
Red meat	1	0.53	123	1.00	1.00
	2	0.89	112	0.92 (0.71–1.18)	0.92 (0.71–1.19)
	3	1.21	115	0.92 (0.72–1.19)	0.95 (0.73–1.23)
	4	1.57	98	0.80 (0.61–1.04)	0.82 (0.62–1.09)
	5	2.22	98	0.80 (0.62–1.05)	0.86 (0.64–1.16)
				*P *for trend = 0.09^b^	*P *for trend = 0.35^b^
Poultry	1	0.09	76	1.00	1.00
	2	0.14	134	0.92 (0.69–1.22)	0.91 (0.69–1.21)
	3	0.22	89	0.84 (0.62–1.14)	0.86 (0.63–1.17)
	4	0.33	97	0.89 (0.66–1.21)	0.94 (0.69–1.28)
	5	0.48	150	1.16 (0.88–1.54)	1.17 (0.88–1.55)
				*P *for trend = 0.06^b^	*P *for trend = 0.06^b^
Fish	1	0.07	106	1.00	1.00
	2	0.13	125	0.94 (0.73–1.22)	0.94 (0.73–1.23)
	3	0.17	92	1.02 (0.76–1.37)	1.09 (0.81–1.47)
	4	0.25	114	0.95 (0.71–1.26)	1.06 (0.80–1.40)
	5	0.44	109	0.99 (0.75–1.30)	0.96 (0.72–1.26)
				*P *for trend = 0.90^b^	*P *for trend = 0.88^b^

In all, 82,063 women were included and there were 546 incident cases of rheumatoid arthritis (RA). Diet was assessed in 1980, 1984, 1986, 1990, 1994, and 1998. Nutrient intakes were cumulatively updated over follow up. ^a^The multivariate models were adjusted for age, total energy intake, body mass index, smoking, and total lifetime breastfeeding. ^b^All *P *values for trend were calculated with median intake of each food in each quintile as a continuous variable. CI, confidence interval.
